# Directional protection scheme using impedance approach for transmission lines

**DOI:** 10.1038/s41598-025-06609-2

**Published:** 2025-06-20

**Authors:** Ahmed R. Adly, Mahmoud A. Elsadd, Mahmoud M. Elgamasy, Omar F. Fadl, Mohamed A. Tolba

**Affiliations:** 1 Nuclear Search Center, Egyptian Atomic Energy Authority, Cairo, Egypt; 2 Electrical Engineering Department, Faculty of Engineering, Damanhour University, Damanhur, Egypt; 3 Electrical Engineering Department, Faculty of Engineering, Menoufia University, Shibīn al Kawm, Egypt; 4 Hot Laboratories Center, Egyptian Atomic Energy Authority, Cairo, Egypt

**Keywords:** Directional relay, Digital protection, Z-matrix, Transmission lines, Electrical and electronic engineering, Energy grids and networks

## Abstract

Identifying the direction of fault is an essential mission of the transmission line protective scheme. This paper discusses a direction protective technique based on a positive impedance approach. The samples of the instantaneous positive sequence voltage component and the instantaneous positive sequence current component are used to determine the impedance approach and complete the Z matrix values. The fault direction can be determine using Z matrix values. Different configurations of the power system are utilized to examine the proposed protective scheme. Software ATP-EMTP (Simulation experiments) verified the feasibility of the presented scheme. To verify the capability and accuracy of presented scheme, it is examined by many fault scenarios such as high fault resistance, far end fault, cross-country fault, power flow change, single pole tripping, CT saturation and noise impact. In addition, the validity of the presented scheme is compared with other protection directional schemes.

## Introduction

The continuity of the power supply is ensured by ensuring that the efficiency of the protection of the power system is at the level of the mark. As a result of the ever-increasing reliance on electrical energy, interruption power outages can significantly deteriorate social and financial stability. Thus, normal power system events need to be reliably ignored from the threat list, which should not be treated as a fault condition^1^. The direction protection scheme for TL is the most thought-provoking of the many schemes developed to protect various power system components. This appears from the fact that for a TL with a stretch of hundreds of kilometers, it is important to estimate the data available on both ends of the line^2^.

The protection of dual circuit lines is very complex due to the effect of mutual coupling, cross-country faults, far end faults evolving faults. High fault resistance (HFR), and cross-country faults^3,4^. This can be a very serious problem in important circuits as system stability is essential. Multi-location and developing effect on the behavior of the protection direction scheme^5^. In^6^, the basic features of distance and direction relays were discussed, as were the potential issues they may encounter in their designs and applications.

In^7^, a scheme for determining the direction of fault was proposed based on the superimposed voltage and superimposed current of the transmission line (TL). The basic features of direction relays were discussed, based on positive sequence current using discrete wavelet transform (DWT). In^9^, a scheme for determining the direction of fault was proposed based on general garden transformation (GPT). In^10^, and^11^, the schemes relied on the current signal only to detect the direction of the fault using the fault signal, also before the fault occurs no phasor estimate required. In^12^, a scheme for determining the direction of fault was proposed using magnitude change in voltage and current signals. In^13^, a scheme for determining the direction of fault was proposed based on fine state machine (FSM) positive sequence current. In^14,15^, the discussed protection technique is based on incorporating Discrete Fourier Transform (DFT) besides artificial neural network (ANN). The DFT is used to determine fundamental voltage and current magnitudes and then the output from DFT is fed to ANN to detect the fault direction. In^16^, the presented protection scheme is based on the combination of DFT and adaptive neuro-fuzzy inference system (ANFIS). This scheme depends only on current signals. However, the schemes^13–16^ depend upon huge samples and training for experience illustration, and also cannot treat the uncertainty considerations in TL.

In^17,18^, a scheme for determining the direction of fault was proposed based on superimposed current component. In^19,20^, a scheme for determining the direction of fault was proposed based on superimposed positive sequence component (SPSC) by contrasting and comparing the angle difference between the voltage and the current signals. In^21^, the discussed scheme relied on the active and reactive power measured at the relay point, also the relay performance was examined during single pole tripping (SPT) in^22^. This scheme is based on apparent powers. In^23^, a scheme for determining the direction of fault was proposed based on smallest square (LS) method to determine the required parameters. But this scheme affected by DC components, noise, and harmonics.

In^24^, a scheme for determining the direction of fault was proposed based on four classifiers (positive and negative sequence components of voltages and currents) and then combined these four classifiers using a fuzzy decision method to estimate the direction of the fault. In^25^, the protection direction scheme provided for the double-circuit TL circuit relied on four classifiers (positive and negative sequence components of voltages and currents). The four classifiers are used to estimate the fault direction. In^26^, the protective scheme provided for the double-circuit TL relied on incorporating a support vector machine (SVM) and discrete wavelet transform (DWT). In^27^, a scheme for double circuit was proposed to determine the direction of the fault using principal component analysis (PCA) of angles between negative and positive sequence components (PSC). In^28^, the protective scheme provided for the double-circuit TL relied on four classifiers (positive voltage sequence components and currents), and these four classifiers are an integrated fuzzy logic (FL) technique to get the fault direction. In^29,30^ the authors proposed the case of the single pole tripping (SPT) state.

In^31^, a technology based on the integration of both DFT and FL was introduced, since FL avoids connection link requirements. In^32^, a current-only technique was introduced using S-Transform to get the angle difference to determine the direction of the fault. In^33^, a scheme for determining the direction of fault was proposed based on angle difference between the post-fault current and the proposed reference signal. In^34^, a scheme for determining the direction of fault was proposed based on the correlation coefficient also using the traveling wave. In^35^, a negative sequence reactive power technique is presented to get the fault direction. In^36^, a superimposed impedance technique is presented based on instantaneous voltage and current samples to get the fault direction. In^37^, a scheme for determining the direction of fault was proposed based on negative sequence- current signals superimposed component, but this scheme affected by HFR as reported in^38^. In^39^ a scheme that relies on negative sequence superimposed impedance was presented as well as illustrating the impact of renewable energy inverter interface generators on directional relays. In^40^, a scheme that relies on fault and pre-fault current samples as well as knowledge of the direction of power flow was presented to get the fault direction. In^41^, a protection scheme was presented for both transmission and distribution systems based on superimposed positive sequence components of current. In^31^, a scheme based on DFT and a fuzzy logic system were introduced. The fuzzy logic system is utilized to escape connection link requirements. In^42^, a scheme for determining the direction of fault was proposed based on angle difference between the fault and the phase of the positive sequence current before the fault was introduced and applied to series-compensated transmission lines during SPT condition. In^43^, a principle was introduced for the construction of a directional element using transfer and consumption characteristics of high-frequency transient energy. This directional element only uses high-frequency transient fault in early-stage which can reliably discriminate the fault direction.

In^44^, the authors developed a directional detection scheme for unbalanced faults in such networks using superimposed symmetrical sequence quantities. The phase angle of the superimposed negative sequence admittance is used to determine fault direction. In^45^, an artificial neural network-based method for precise fault distance and direction estimate is given for the protection of transmission lines. The proposed method uses the voltage and current available at only the local end of line. In^46^, the line-mode fault component power based directional pilot protection is proposed for flexible DC distribution grid. the line-mode fault component power is built as the directional criterion. In^47^, the fault direction characteristics are constructed using the wavelet energy entropy difference of the fault currents of the two lines. The fault direction characteristics are combined with the start fault phase angle and transition resistance.

In^48^, the S-transform is employed to extract the time–frequency information of the fault transient voltage components, the direction identification criterion based on the energy difference between the reverse and forward transient voltage components is constructed. In^49^, the paper studies the characteristics of negative sequence power direction when the measuring points of the main line and branch lines of DG distribution network in forward fault or backward fault under the negative sequence current suppression strategy and puts forward a multi-terminal directional. In^50^, A directional relaying method is proposed for such connectivity using the phase angle difference between positive sequence voltage and the line current measured at the local bus relay.

In case of cross-country and evolving faults the direction of fault avoids maloperation of the relay as reported in^51^. Also, many techniques have a bad response due to mutual coupling in double-circuit TL. In this paper, a new protection technique based on instantaneous current and voltage samples is introduced to accurately estimate the fault direction to achieve the speedy and correct tripping, select the appropriate impedance element to locate faults, enable the single-pole operation and thus increase system stability. The proposed protection scheme (PPS) includes a standard for detecting fault direction and eliminating shortcomings and many issues related to previous protection schemes where its scheme works correctly in the case of SPT, inter-circuit fault, HFR, near internal fault, variation in operating voltage, variation in operating frequency, variation in source impedance, difference in source strength as well as power flow change. Table 1 reviews the transitional aspects of different protection techniques to get the direction of fault in TLs.

The rest of the paper is arranged as follows: A brief of the direction protection implementation challenges in TL are detailed in Sect. 1. The proposed protection scheme for TL is described in Sect. 2. Numerous simulation cases using the ATP program are implemented in Sect. 3 to validate the suggested method. A discussion of the generalization of the WSCC 9 bus system in Sect. 4. a comparison between PPS and other conventional techniques stated in the literature in Sect. 5. Finally, the conclusion is provided in Sect. 6.

## Proposed protection scheme description

### Fault analysis

**Fig. 1:**
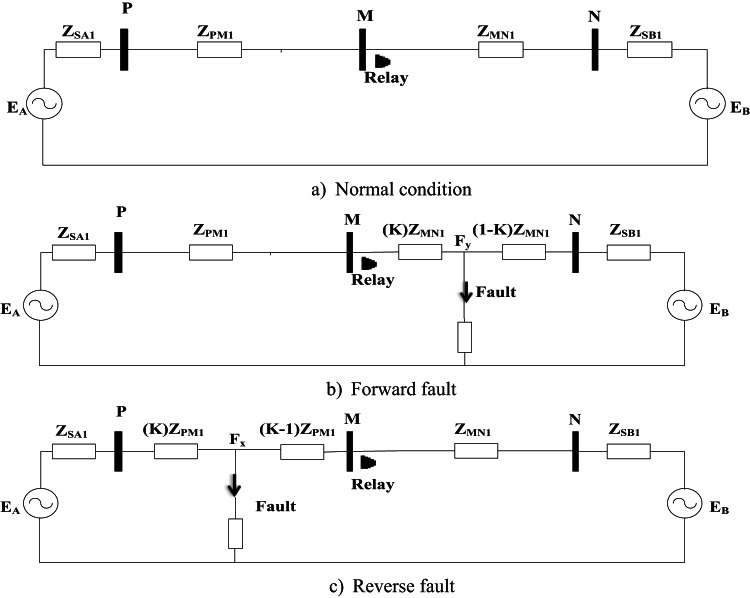
Equivalent circuit for positive sequence network.

The proposed directional protection detection algorithm is analyzed by representing a simple circuit consisting of two power lines and three buses as shown in Fig. 1.a, considering Z_PM1_ and Z_MN1_ are the positive sequence resistance of the PM and MN lines, respectively. The proposed relay located in bus M and the direction power flow from bus P to bus N. Determine the positive sequence components based on Eqs. (1) and (2).

1 $$\:{I}_{1}=\frac{{I}_{a}+{\alpha\:I}_{b}+{{\alpha\:}^{2}I}_{c}}{3}$$

2 $$\:{V}_{1}=\frac{{V}_{a}+{\alpha\:V}_{b}+{{\alpha\:}^{2}V}_{c}}{3}$$

$$\:\text{W}\text{h}\text{e}\text{r}\text{e},\:\alpha\:={e}^{j120},\:{\alpha\:}^{2}={e}^{j240}$$

The voltage PSC at the relay location can be determined as follows:

3 $$\:{\text{V}}_{1}^{\text{p}\text{r}\text{e}}=\:{\text{E}}_{\text{A}}-{\text{Z}}_{\text{P}\text{M}1}{\text{I}}_{1\:\text{p}\text{r}\text{e}}$$

the PSC voltage at the relay is as follows, when the power flow changed from bus N to bus P

4 $$\:{\text{V}}_{1}^{\text{p}\text{r}\text{e}}=\:{\text{E}}_{\text{B}}-{\text{Z}}_{\text{M}\text{N}1}{\text{I}}_{1\:\text{p}\text{r}\text{e}}$$

Equations (3), (4) provide PSC voltage before fault condition.

When a shunt fault on the forward side appears at a distance k from bus M, the positive sequence circuit is changes from Fig. 1a,b, and then, PSC voltage is as follows:

**Table 1:** Comparative study of different protection schemes for the fault direction on TL.

Technique Item	Voltage (kV)	Data Input	Single / Double Circuit	Measured data at Terminals	Used Scheme	Calculation Features	HFR	PFC Detect	S_f_ (kHz)
Ref.^7^	735	Current & Voltage	Single circuit only	One	DFT	Fault current and voltage components	Not Tested	Need	9.6
Ref.^8^	500	Current only	Double circuit only	Two	DWT	Positive sequence current coefficients	Not Tested	Need	200
Ref.^9^	500	Current & Voltage	Single circuit only	One	GPT	Negative sequence reactance	Tested	Need	1
Ref.^10^	N.R	Current only	Single circuit only	One	N.R	superimposed component of the current	Not Tested	Need	N.R
Ref.^11^	30 & 20	Current only	Distribution system	One	DFT	Post-fault current component	Tested	No Need	1
Ref.^12^	400	Current & Voltage	Single circuit only	One	DFT	Positive-sequence current and voltage components	Tested	Need	1
Ref.^13^	400	Current only	Single circuit only	One	FSM	positive sequence current component	Tested	No Need	N.R
Ref.^14^	400	Current & Voltage	Single circuit only	One	DFT & ANN	Fault voltage and current components	Tested	Need	1
Ref.^15^	400	Current & Voltage	Single circuit only	One	DFT & ANN	Fault voltage and current components	Tested	Need	1
Ref.^16^	400	Current only	Single circuit only	One	DFT & ANFIS	Post-fault current component	Tested	No Need	1
Ref.^17^	500	Current only	Single circuit only	Two	CM	Fault current & pre-fault current	Tested	Need	0.8
Ref.^18^	20 & 230	Current only	Single circuit only	One	DFT	Imaginary part of post-fault current	Tested	Need	10
Ref.^19,20^	500	Current & Voltage	Single circuit only	One	DFT	Positive-sequence current and voltage components	Not Tested	Need	0.8
Ref.^21^	11 & 525	Current & Voltage	Single & double circuit2	One	N.R	Active & reactive power	Tested	Need	N.R
Ref.^22^	400	Current & Voltage	Single & double circuit2	One	N.R	Apparent powers	Tested	Need	1
Ref.^23^	400	Current & Voltage	Single circuit only	One	LST	Line parameters estimating	Tested	Need	1
Ref.^24^	400	Current & Voltage	Double circuit only	One	LST & FL	Four Indices (Ind_1_-Ind_3_)	Tested	Need	1
Ref.^25^	400	Current & Voltage	Double circuit only	One	LST & VT	Four Indices (Ind_1_-Ind_3_)	Tested	Need	1
Ref.^26^	400	Current	Double circuit only	Two	DWT & SVM	Fault current component	Tested	Need	1
Ref.^27^	400	Current & Voltage	Double circuit only	One	PCA & LST	Three Indices (Ind_1_-Ind_3_)	Tested	Need	1
Ref.^28^	400	Current & Voltage	Double circuit only	One	LST & FL	Four Indices (Ind_1_-Ind_3_)	Not Tested	Need	1
Ref.^29^	400	Current	Single & double circuit2	One	DFT	Superimposed positive sequence current	Tested	Need	1.6
Ref.^30^	400 & 100	Current & Voltage	Single & double circuit2	Two	DFT	Superimposed negative sequence component	Tested	Need	1.6
Ref.^31^	735	Current & Voltage	Single circuit only	One	DFT & FL	Positive-sequence current and voltage components	Tested	Need	1.2
Ref.^32^	13.8 & 25	Current	Distribution system	One	S-Transform	Fault current & pre-fault current	Not Tested	No Need	N.R
Ref.^33^	NR	Current	Double circuit only	One	N.R	Fault & pre-fault current	Not Tested	No Need	N.R
Ref.^34^	750	Current & Voltage	Single circuit only	One	TW	Incremental quantities	Tested	No Need	10
Ref.^35^	25	Current & Voltage	Distribution system	One	N.R	Reactive power	Tested	Need	N.R
Ref.^36^	33	Current & Voltage	Distribution system	One	LST	Superimposed impedance	Tested	No Need	1
Ref.^37^	400	Current	Single & double circuit2	One	DFT	NSSC current	Not Tested	Need	1
Ref.^38^	400	Current & Voltage	Single circuit only	One	DFT	NSSC current and voltage	Tested	Need	1

5 $$\:{\text{V}}_{1}^{\text{f}}=\:{\text{E}}_{\text{A}}-{\text{Z}}_{\text{P}\text{M}1}{\text{I}}_{1\:\text{f}}$$

By subtracting (1) from (5), the resultant superimposed PSC voltage is as follows:

6 $$\:{V}_{1}^{{\prime\:}}=\:-{\text{Z}}_{\text{P}\text{M}1}{I}_{1}^{{\prime\:}}$$

where $\:{I}_{1}^{{\prime\:}}\:\text{a}\text{n}\text{d}\:\:\:{V}_{1}^{{\prime\:}}\:$ are the superimposed PSC current and voltage at the fault, respectively.

If the shunt fault happens on the inverted side (between bus P and bus M) as shown in Fig. 1c, then, we have:

7 $$\:{\text{V}}_{1}^{\text{f}}=\:{\text{E}}_{\text{B}}-{\text{Z}}_{\text{M}\text{N}1}{\text{I}}_{1\:\text{f}}$$

Subtracting (2) from (7):

8 $$\:{V}_{1}^{{\prime\:}}=\:-{\text{Z}}_{\text{M}\text{N}1}{I}_{1}^{{\prime\:}}$$

Equations (8) and (6) can be utilized to estimate the path of the fault. This equation can be written in the following form (sampled data) as follows:

9 $$\:\left[\begin{array}{c}\begin{array}{c}{V}_{1}^{{\prime\:}}\:\left(\text{K}\right)\\\:{V}_{1}^{{\prime\:}}\:(\text{K}+1)\\\:{V}_{1}^{{\prime\:}}\:(\text{K}+2)\end{array}\\\:----\\\:{V}_{1}^{{\prime\:}}\:(K+n)\end{array}\right]=\left[\begin{array}{c}\begin{array}{c}{I}_{1}^{{\prime\:}}\:\left(\text{K}\right)\\\:{I}_{1}^{{\prime\:}}\:(\text{K}+1)\\\:{I}_{1}^{{\prime\:}}\:(\text{K}+2)\end{array}\\\:----\\\:{I}_{1}^{{\prime\:}}\:(K+n)\end{array}\right]\text{*}\:{\left[\text{Z}\right]}_{1\text{*}1}$$

where k = 1, 2, …n shows the samples of signals and n shows the number of samples in a period. In case of 1.6 kHz as a sampling frequency, we get 64 samples in a period and Eq. (9) can be as follow:

10 $$\:{\left[\text{Z}\right]}_{1\text{*}1}={\left[\begin{array}{c}\begin{array}{c}{I}_{1}^{{\prime\:}}\:\left(\text{K}\right)\\\:{I}_{1}^{{\prime\:}}\:(\text{K}+1)\\\:{I}_{1}^{{\prime\:}}\:(\text{K}+1)\end{array}\\\:----\\\:{I}_{1}^{{\prime\:}}\:(K+n)\end{array}\right]}^{-1}*\left[\begin{array}{c}\begin{array}{c}{V}_{1}^{{\prime\:}}\:\left(\text{K}\right)\\\:{V}_{1}^{{\prime\:}}\:(\text{K}+1)\\\:{V}_{1}^{{\prime\:}}\:(\text{K}+2)\end{array}\\\:----\\\:{V}_{1}^{{\prime\:}}\:(K+n)\end{array}\right]$$

Based on the previous analysis, the above matrix can be used to determine the fault direction. If a fault occurs in the forward side, the same matrix values will be positive, however the matrix values will be negative for the faults in the backward side.

###  Direction protection scheme

**Fig. 2:**
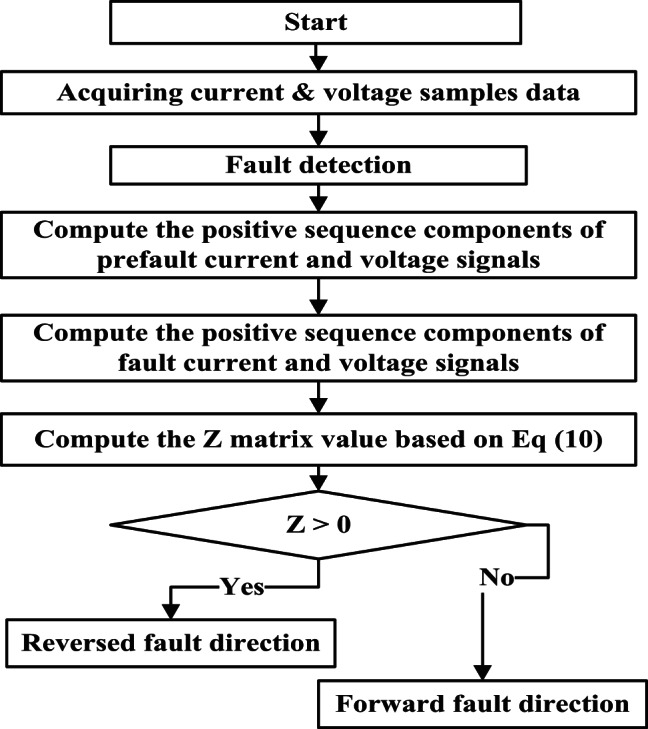
Proposed directional protection detection algorithm.

Figure 2 shows the proposed directional protection detection algorithm that is not utilized as a fault detector, however, the scheme for overcurrent is utilized for this function as per “IEC 60255”^21^. Several directional protection detection schemes based on voltage and current signals for the conventional TL^22,23^. Its schemes for directional relaying have limitations for changing the power flow, far-end fault, single pole tripping, cross-country fault, and inter-circuit fault leading to mal-operation. The proposed directional protection detection algorithm is based on the data samples (one cycle window) of PSC of voltage and current. Then these data are utilized to estimate the Z matrix. The fault direction is detected by utilizing the Z matrix values. The relation between the PSC of current, Z matrix, and PSC voltage are explained in Eq. (11).

11 $$\:{\left[\text{Z}\right]}_{1\text{*}1}={\left[{I}_{1}^{{\prime\:}}\right]}^{-1}\text{*}\left[{V}_{1}^{{\prime\:}}\right]$$

Therefore, the proposed directional protection detection algorithm defines the voltage and current samples and then calculates the value of the Z matrix to obtain the relay decision. The action for the proposed directional protection detection algorithm at bus M is:

- If the Z matrix value < K1 →► Reverse direction of the fault.
- If the Z matrix value > K2 →► Forward direction of the fault.

In real life about that any scheme in general for protection, need a starting method to avoid false tripping due to very low values than can have noise, that is the reason manufacturers provide always threshold values. So we will use K1 and K2 for detect the fault direction.

Where, K1 is the reliable coefficient with a range of + (0.3–0.4) for reverse faults and K2 is the reliable coefficient with a range of − (0.3–0.4) for forward faults.

In the fault case during SPT condition, (V_SPT1_) is non-affected but (I_a_=0) owing to the existence of SPT in phase-a. Then, (I_SPT1_) throughout SPT can be calculate as in Eq. (12). Consequently, the proposed scheme will get correct action.

12 $$\:{I}_{SPT1}=\frac{{\alpha\:I}_{b}+{{\alpha\:}^{2}I}_{c}}{3}$$

If a three-phase shunt fault occurs, the three-phase voltage signals will be very low and can reach zero at a bolted fault at the relay point. Consequently, getting the Z matrix value is very difficult, to avoid this scenario an adaptive memory can be used.

**Fig. 3:**
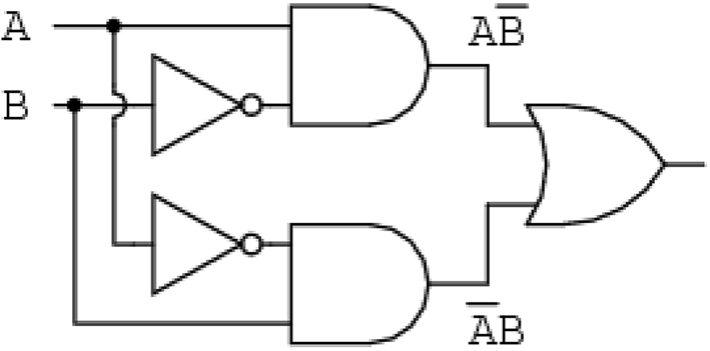
Proposed directional protection detection algorithm logic circuit.

Figure 3 declares the logic circuit of the PPS. A and B are the inverse matrix of the superimposed positive sequence component current and matrix of the superimposed positive sequence component voltage, respectively.

A = 0, 1 indicates the inverse matrix of the superimposed positive sequence component current is positive or negative value.

B = 0, 1 indicates the matrix of the superimposed positive sequence component voltage is positive or negative value.

Table 2 show the truth table of the proposed directional protection detection algorithm, and Eq. 13 describe the logic equation of the proposed directional protection detection algorithm. Consequently, the result = 0 denotes reverse fault and the result = 1 denotes forward fault.

13 $$\:f=A\bullet\:{B}^{-}+{A}^{-}\bullet\:B$$

**Table 2:** Proposed directional protection detection algorithm truth table.

A	B	Results
1	1	0
1	0	1
0	1	1
0	0	0

### Computational requirement time

If the fault occurs, we notice that the relay seen it, and then the detection algorithm determines the initial wave front by processing this signal by converting it to phase mode to obtain positive sequence components. The value of the Z matrix across a moving window with a length of one cycle is determined. The proposed directional protection detection algorithm determines the fault direction in less than 0.02 s. In practice, a microprocessor-based relay with a performance of 1 GFLOPS performing signal processing and detection at the fault direction takes approximately 7 and 10 µs^52^.

Comparing the proposed directional protection detection algorithm with the ABB commercial directional protection relay (RXPDK 2 H and RAPDK)^53^. In ABB the setting range time is adjusted by the relay. The first one is definite time with a time delay from 0.05 to 8.1 s, and the second one is inverse time with a time delay from 0.05 to 1.1 s. Therefore, PPS takes a total operating time of about 0.0201 s. This small time verifies the fast performance.

## PPS performance

### Scenarios studied

**Fig. 4:**
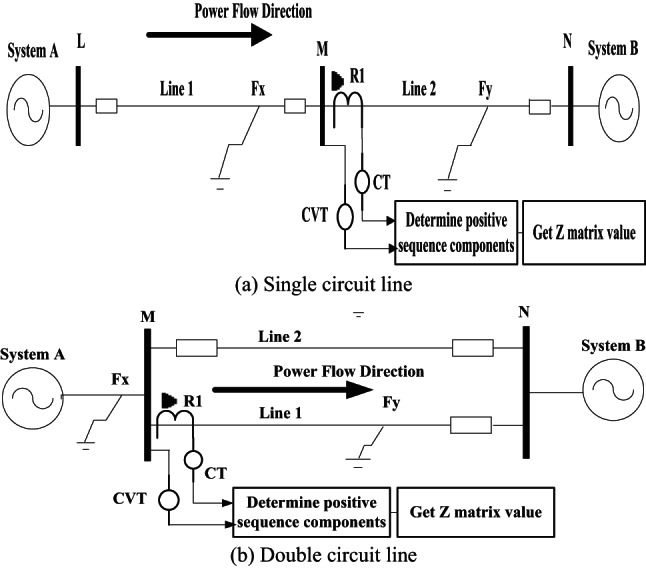
Sample power system and ATP application photo.

Several simulations have been accomplished using ATP/EMTP for the evaluation of the proposed protection algorithm. Figures 4 shows the systems under study. Figure 4a shows the single circuit line^29^, and Fig. 4b shows the double circuit line^30^. The overhead line is modelled in non-transposed double-circuited type, where parameters are calculated in 50 Hz. It should be noted that in calculating machine dynamics, phenomena are mostly in power frequency, so power frequency based line parameters are to be applied. Phase line locations are “a”, “b” and “c” from the top in one side, and “c”, “b” and “a” in the other side for obtaining as better symmetry^54^. Proposed directional protection detection algorithm response was examined ten times. The main scenarios examined were created by varying parameters such as the fault location, fault inception time, fault resistance, far-end fault, close-in fault, cross-country fault for double circuit line, SPT, and PFC under dissimilar system situations. Only the main scenarios are clarified in the subsequent sections. The scenarios are consecutively detected the fault direction that can be classified as follows:

#### Scenario 1

Influence of different fault parameters (types, fault location, fault time, fault resistance, load angle) for single circuit line.

#### Scenario 2

HFR for single circuit line.

#### Scenario 3

SPT for single circuit line.

#### Scenario 4

PFC for single circuit line.

#### Scenario 5

Close in fault for single circuit line.

#### Scenario 6

Influence of different fault parameters (types, fault location, fault time, load angle, fault resistance) for double circuit line.

#### Scenario 7

HFR for double circuit line.

#### Scenario 8

SPT for double circuit line.

#### Scenario 9

PFC for double circuit line.

#### Scenario 10

Close-in fault for double circuit line.

#### Scenario 11

Far end fault for double circuit line.

#### Scenario 12

Cross-country fault for double circuit line.

#### Scenario 13

Influence of Noise on the PPS.

#### Scenario 14

Influence of sampling frequency on the PPS.

#### Scenario 15

Influence of alternative in operating voltage, frequency on the PPS.

#### Case 16

Influence of alternative in source impedance on the PPS.

#### Scenario 17

Influence of Source Strength on the PPS.

#### Scenario 18

Influence of different transmission line lengths on PPS.

#### Scenario 19

Influence of CT saturation on PPS.

### Discussion and results

#### Influence of different fault parameters (types, fault location, fault time, fault resistance, load angle) for single circuit line: Scenario 1.

The proposed directional protection detection algorithm behavior in case of different fault parameters (types, fault location, fault time, fault resistance, load angle) for are presented in the subsection. The results of different fault parameters and fault conditions are confirmed in Table 3. It is spotted that the proposed directional protection detection algorithm decision has high precision as per Table 3 under different fault conditions.

**Table 3:** Influence of different fault parameters (types, fault location, fault time, fault resistance, load angle) for a single circuit line (Scenario 1).

The considered conditions					Results	
Fault types	Fault position	Resistance (Ω)	Inception Instant (sec)	Load angle of sources (degree)	Z matrix value	Direction result
ABCG	Forward	3	0.18	11	-4.3	Forward
ABCG	Reverse	2	0.17	5	6.6	Reverse
ABC	Forward	35	0.17	9	-3.9	Forward
ABC	Reverse	40	0.16	8	5.5	Reverse
BC	Forward	16	0.15	12	-4.7	Forward
BC	Reverse	16	0.15	9	5.4	Reverse
AC	Forward	20	0.13	12	-4.2	Forward
AC	Reverse	25	0.16	16	6.8	Reverse
BCG	Forward	20	0.14	8	-3.9	Forward
BCG	Reverse	15	0.17	10	4.5	Reverse
ACG	Forward	35	0.17	5	-4.9	Forward
ACG	Reverse	75	0.15	8	4.6	Reverse
AB	Forward	1	0.18	15	-4.9	Forward
AB	Reverse	2	0.16	5	5.6	Reverse
CG	Forward	3	0.17	12	-4.8	Forward
CG	Reverse	5	0.17	16	4.0	Reverse
ABG	Forward	5	0.18	4	-3.6	Forward
ABG	Reverse	5	0.15	14	6.9	Reverse
AG	Forward	20	0.15	6	-4.8	Forward
AG	Reverse	75	0.17	4	3.9	Reverse
BG	Forward	5	0.14	12	-5.2	Forward
BG	Reverse	5	0.18	5	4.1	Reverse

#### HFR for single circuit line

**Fig. 5:**
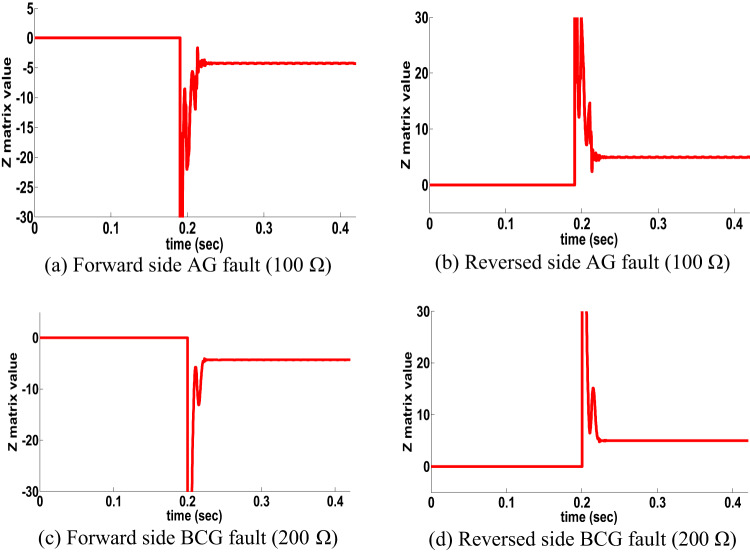
HFR results for single circuit line (scenario 2).

HFR has impact in many protection techniques. an AG fault with a fault resistance of 100 Ω occurred at the time t = 0.18 s. on the front side. Figure 5 shows the dynamics performance of the proposed directional algorithm for the detection of the forward fault (Z matrix being negative). Then the AG fault occurred on the reverse side at the time t = 0.18 s, the dynamics performance of the proposed directional algorithm is shown in Fig. 5 b shows a positive Z matrix value. Figures 5c,d also show the dynamic performance of proposed directional algorithm in the case of a two-sided BCG fault occurring with a fault resistance of 200 Ω. Figure 5 shows that the proposed directional algorithm decision is precision under HFR at a single circuit line. In addition, a large number of scenarios for the healthy conditions of the transmission lines and external faults have been investigated and used in providing protection algorithms.

#### SPT for single circuit line: scenario 3

**Fig. 6:**
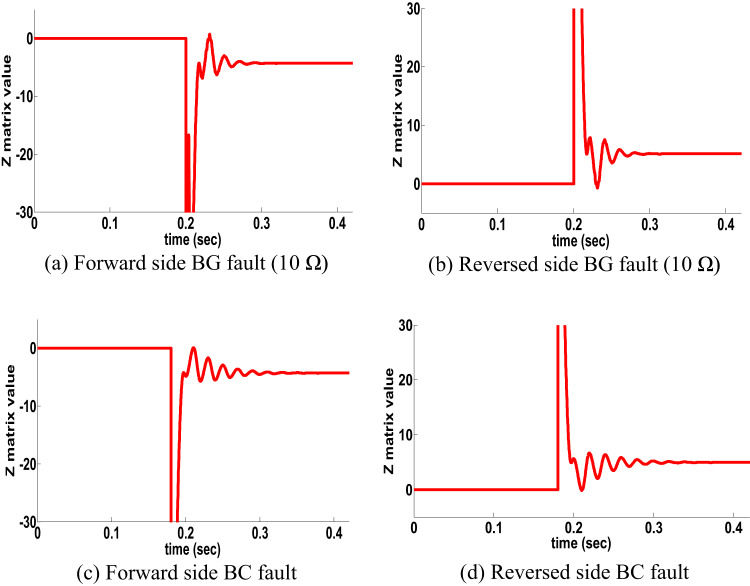
SPT results for single circuit line (scenario 3).

TL availability increasing and enhancement power transfer capacity by using SPT technique. As a result, protection schemes are sensitive to the SPT process. Consequently, an AG fault is assumed to occur in line 2 at the F_y_ position t_1_ = 0.1 S to create SPT case. After that, the SPT is executed by opening the faulted phase. Suppose a BG fault at t_2_ = 0.2 S on the forward side occurs, and the performance of PPS is shown in Fig. 6a In addition, if a BG fault on the reverse side t_2_ = 0.2 S occurred, and the behavior of the proposed scheme is shown in Fig 6b In addition, another test was done to ensure the availability of the proposed scheme. If the BC fault type in two sides occurs as shown in Fig. 6c,d. The results obtained, shown in Fig. 6 of the SPT cases, showed the availability of the proposed directional protection detection algorithm.

####  PFC for single circuit line: scenario 4

Angel of source A and angel of source B changed to examine the power flow change condition in the following equation:

14 $$\Delta \delta {\text{ }} = \delta _{A} - ~\delta _{B}$$

Where ∆δ value (negative or positive) denoted that power flow change (reverse or forward). Note that if the PFC the rules of the proposed directional protection detection algorithm will be the same as in Sect. 3.

**Fig. 7:**
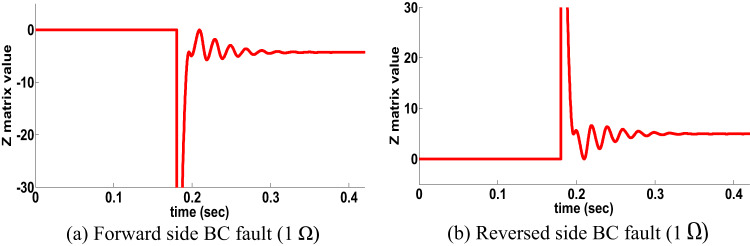
PFC results for single circuit line (scenario 4).

The ∆δ is changed to be negative. To address the proposed scheme behavior under this situation, BC fault (1Ω) at 0.18 s on two sides occurred. Figure 7a shows the performance of the proposed directional algorithm for forward faults. For BC fault reverse side (1Ω) at t = 0.18 s occurred, Fig. 7b shows the performance of the proposed directional algorithm for reverse fault (Z matrix is positive). It is noticed from Fig. 7 that the proposed directional protection detection algorithm decision is accurate under PFC at a single circuit line.

#### Close-in fault for single circuit line: scenario 5

**Fig. 8:**
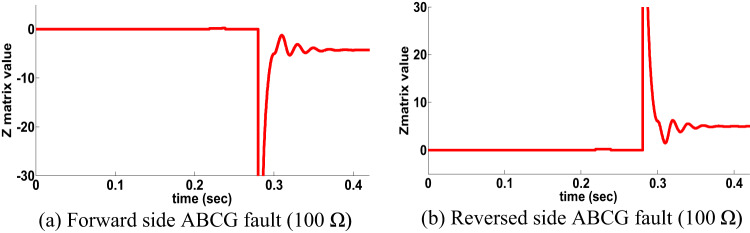
Close in fault results for single circuit line (scenario 5).

ABCG close-in fault occurred with 100 Ω at 0.28 s in the forward side, the performance of the proposed directional algorithm for this case is shown in Fig. 8 (Z matrix is negative). Another fault done in reverse side and the proposed directional protection detection algorithm performance is shown in Fig. 8. It is remarked from Fig. 8 that the proposed directional protection detection algorithm decision is accurate under close-in faults at a single circuit line.

#### Influence of different fault parameters (types, fault location, fault time, load angle, fault resistance) for double circuit line: scenario 6

The proposed directional protection detection algorithm behavior in the case of different fault parameters (types, fault location, fault time, fault resistance, load angle) for double circuit lines are presented in the subsection. The different fault parameters results are demonstrated in Table 4. It is noticed that the proposed directional protection detection algorithm decision has high accuracy in Table 4 under different fault parameters and fault conditions.

**Table 4:** Influence of different fault parameters (types, fault location, fault time, fault resistance, load angle) for double circuit line (Scenario 6).

The considered conditions					Results	
Fault types	Fault position	Resistance (Ω)	Inception Instant (sec)	Load angle of sources (degree)	Z matrix value	Direction result
AG	Forward	10	0.187	4	-1.6	Forward
BG	Forward	5	0.165	7	-2.9	Forward
CG	Forward	30	0.176	11	-2.5	Forward
ABG	Forward	5	0.192	16	-2.2	Forward
ACG	Forward	15	0.165	4	-2.8	Forward
BCG	Forward	30	0.167	7	-1.8	Forward
AB	Forward	2	0.168	11	-3.4	Forward
BC	Forward	1	0.163	16	-2.3	Forward
AC	Forward	2	0.172	4	-1.9	Forward
ABCG	Forward	0.1	0.176	7	-4.2	Forward
ABC	Forward	1	0.179	11	-1.5	Forward
AG	Reverse	15	0.182	16	4.1	Reverse
BG	Reverse	25	0.181	4	2.7	Reverse
CG	Reverse	20	0.183	7	3.9	Reverse
ABG	Reverse	10	0.165	11	4.6	Reverse
ACG	Reverse	1	0.164	16	5.1	Reverse
BCG	Reverse	0.5	0.176	4	5.6	Reverse
AB	Reverse	2	0.175	7	4.8	Reverse
BC	Reverse	1	0.184	11	4.6	Reverse
AC	Reverse	2	0.182	16	3.9	Reverse
ABCG	Reverse	30	0.169	7	2.8	Reverse
ABC	Reverse	2	0.173	11	3.7	Reverse

#### HFR for double circuit line: scenario 7

**Fig. 9:**
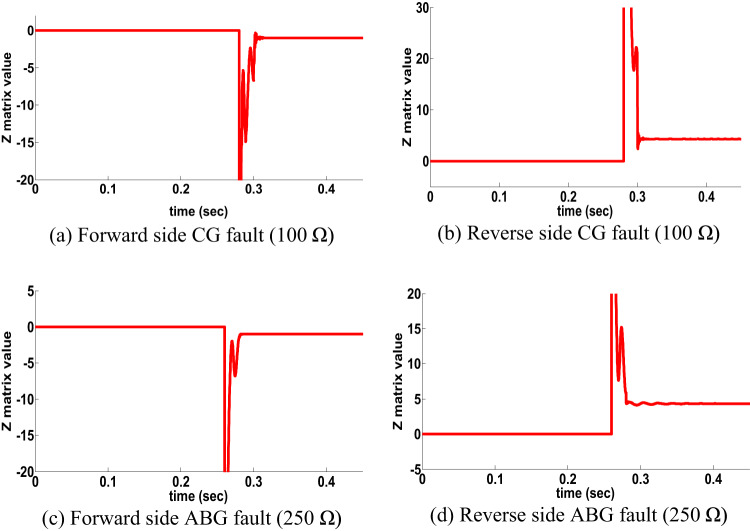
HFR results for double circuit line (scenario 7).

The behavior of the proposed directional protection detection algorithm is examined for HFR such as CG fault occurring with 100 Ω and ABG fault occurring with 250 Ω fault resistance. CG fault at the forward side occurred, the behavior of the proposed directional algorithm (Z matrix is negative) is shown in Fig. 9a. Then the same fault occurred at reverse side. The behavior of the proposed directional algorithm (Z matrix is positive) is shown in Fig. 9b. Then, ABG fault occurred at the forward and revers sides with 250 Ω fault resistance. Figures 9c,d show the performance of the proposed directional protection detection algorithm in scenario of ABG fault occurring on two sides with 250 Ω fault resistance. It is remarked from Fig. 9 that the proposed directional protection detection algorithm decision is accurate under HFR at the double circuit line.

#### SPT for double circuit line: scenario 8

**Fig. 10:**
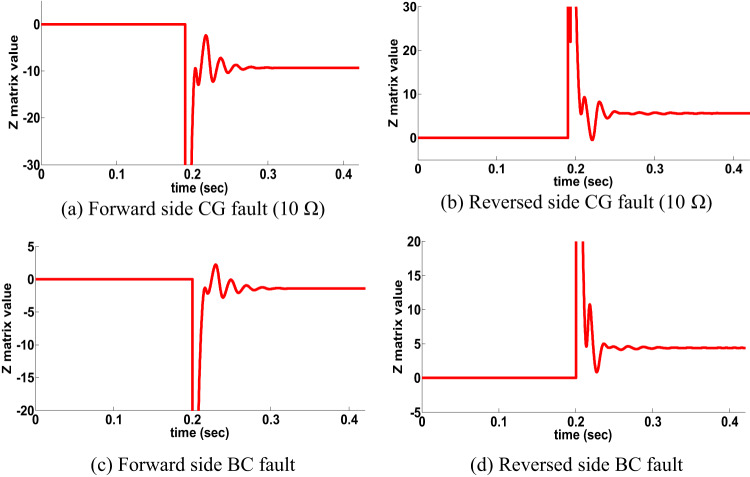
SPT results for double circuit line (scenario 8).

The behavior of the proposed directional protection detection algorithm is examined for SPT for double circuit line such as AG fault occurs in line 2 (position Fy, t_1_ = 0.1 S) then opening the phase faulted, and create CG fault (t_2_ = 0.18 S) on the forward side. The behavior of the proposed directional algorithm is shown in Fig. 10.a. Then the same fault scenario occurred at reverse side. The behavior of the proposed directional algorithm is shown in Fig. 10b. Another scenario was done a BC fault type occurred at the forward and revers sides as shown in Fig. 10c & d. It is remarked from Fig. 10 that the proposed directional protection detection algorithm decision is accurate under SPT at the double circuit line.

#### PFC for double circuit line: scenario 9

**Fig. 11:**
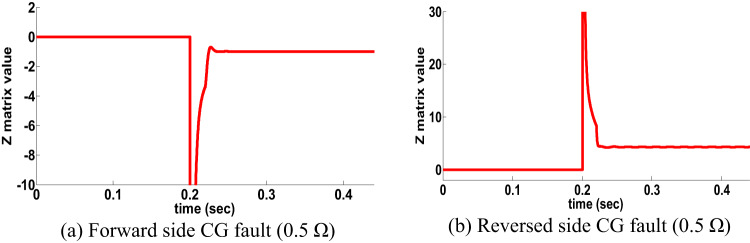
PFC results for double circuit line (scenario 9).

To examine the PFC situation in case of double circuit line is the same for single circuit line by using Eq. (14). ∆δ is changed to be -ve. To explain the behavior of the proposed directional protection detection algorithm, CG fault happened (0.2 S in different sides with 0.5 Ω) in different sides. Figures 11a,b show the performance of the proposed directional protection detection algorithm in scenario of PFC.

####  Close in fault for double circuit line: scenario 10

**Fig. 12:**
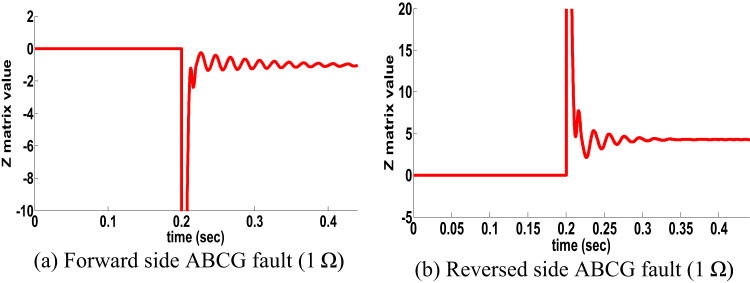
Close in fault results for double circuit line (scenario 10).

The proposed directional protection detection algorithm is examined for 3-φ close-in fault occurred at 0.2 s in two sides with 1 Ω, the proposed directional protection detection algorithm behavior for faults at both sides is shown in Fig. 12. It is remarked from Fig. 12 that the proposed directional protection detection algorithm decision is precise under close in faults in double circuit line.

#### Far end fault for double circuit line: scenario 11

**Fig. 13:**
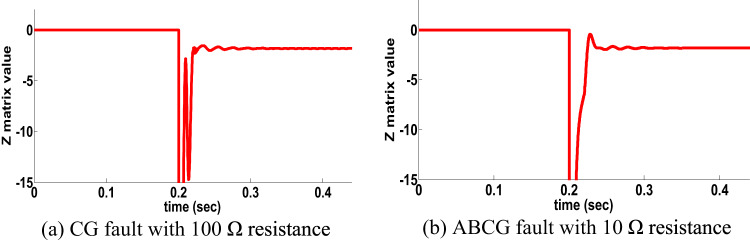
Far end faults results for double circuit line (scenario 11).

The proposed directional protection detection algorithm is examined for far end faults close to the remote bus N. Usually the conventional protection schemes based on travelling wave (TW) are unable to detect the far end fault. Dynamic behavior of the proposed directional protection detection algorithm at this scenario is evaluated with different fault types. If SLG (CG) fault happened at 0.2 S, the PPS behavior is shown in Fig. 13a declare the Z matrix value of the proposed directional protection detection algorithm is -ve. If ABCG fault happened at time t = 0.2 S, the proposed directional protection detection algorithm behavior is shown in Fig. 13b declare the Z matrix value is -ve. It is spotted from Fig. 13 that the proposed directional protection detection algorithm decision is precise under far end faults.

#### Cross country fault for double circuit line: scenario 12

**Fig. 14:**
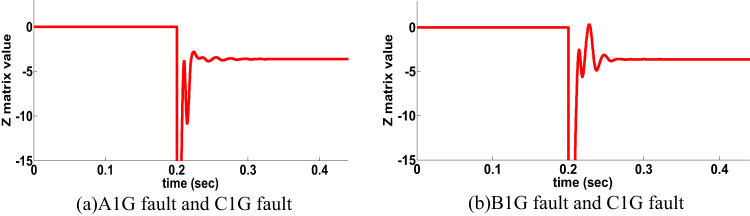
Cross-country faults result for double circuit line (scenario 12).

Cross-country fault occurs when SLG faults happens in different lines of the same circuit in two different positions. The proposed directional protection detection algorithm is tested for this case with different fault position and location. Table 5 declare the scenario results of proposed directional protection detection algorithm. Also Fig. 14 show cross-country faults in time domain. It is noticed from the Table 5; Fig. 14 that the proposed directional protection detection algorithm decision is accurate under cross-country faults.

**Table 5:** Cross-country faults test results for double circuit line (scenario 12).

The considered conditions						Results	
First fault	Second fault	Fault position (km)		Resistance (Ω)		Z matrix value	Direction result
		First fault	Second fault	First fault	Second fault		
A1G	B1G	10	50	100	20	-3.5	F
A1G	C1G	80	10	5	10	-4	F
B1G	A1G	20	100	1	100	-3.8	F
B1G	C1G	90	20	5	25	-4.1	F
C1G	B1G	30	90	15	120	-2.9	F
C1G	A1G	100	10	150	1	-3.1	F

#### Influence of noise on the PPS: scenario 13

**Fig. 15:**
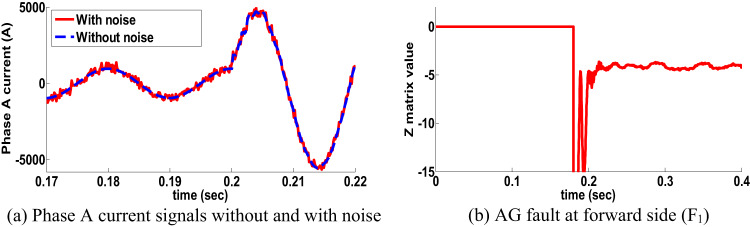
Behavior of PPS under incorporated the noise (scenario 13).

The behavior of the proposed directional protection detection algorithm has been tested during noisy conditions. A signal-to-noise ratio (SNR) of 60–30 dB has been studied. The AG fault in single circuit line with 10 Ω at forward side was tested to achieve the noise case. Phase A current signals without and with noise is shown in Fig. 15 shows the performance of the proposed directional protection detection algorithm for this situation. Consequently, the proposed directional protection detection algorithm is not affected by the existence of noise.

#### Influence of sampling frequency on the PPS: scenario 14

**Fig. 16:**
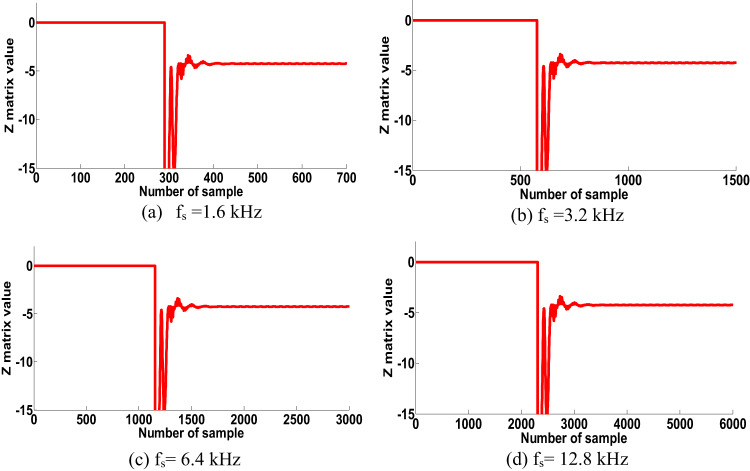
PPS performance under different sampling frequency (scenario 14).

To examine the impact of sampling frequency, the proposed directional protection detection algorithm under CG fault occurred at forward side in single circuit line by changing the current sampling frequency. A CG fault has occurred at forward side is generated. Figure 16 demonstrate the proposed directional protection detection algorithm behavior under dissimilar sampling frequencies.

#### Influence of variation in operating voltage, frequency on the PPS: scenario 15

The proposed directional protection detection algorithm is tested for single circuit line under changing the operating frequency and voltage. In this scenario, +/-10% margin is utilized for voltage dissimilarity and +/-0.5% margin is utilized for the frequency difference. In this case, Table 6 shows the scenario results that declare and confirms the robustness and effectiveness of the proposed directional protection detection algorithm, respectively.

**Table 6:** Changing the voltage and frequency PPS behavior (scenario 15).

The conditions studied					Results	
Frequency (Hz)	Voltage (kV)	Fault types	Fault position	Resistance (Ω)	Z matrix value	Direction result
50.5	385	AG	F	30	-2.5	F
52.0	385	AG	R	2.5	3.6	R
52.0	395	BG	F	25	-3.8	F
47.0	385	BG	R	23	2.7	R
47.0	415	CG	F	2	-3.6	F
50.5	425	CG	R	8	4.3	R
52.0	415	ABG	F	45	-4.5	F
52.0	415	ABG	R	15	3.2	R
48.5	380	ACG	F	10	-1.8	F
48.5	370	ACG	R	15	4.6	R
50.5	390	BCG	F	35	-2.5	F
50.5	390	BCG	R	5	5.4	R
470	495	AB	F	15	-4.6	F
52.0	495	AB	R	25	3.3	R
50.5	400	BC	F	15	-2.8	F
48.5	400	BC	R	5	2.7	R
52.0	415	AC	F	45	-3.9	F
50.5	415	AC	R	10	1.7	R
48.5	385	ABCG	F	5	-3.6	F
52.0	395	ABCG	R	5	3.9	R
50.5	415	ABC	F	40	-2.9	F
48.5	415	ABC	R	45	3.7	R

#### Influence of dissimilarity in source impedance on the PPS: scenario 16

Proposed directional protection detection algorithm is examined for single circuit line as consider dissimilarity of the source impedance. In this scenario, +/-10% margin is utilized for variation of source impedance. In this scenario, Table 7 show the results that declare and confirms effectiveness of the proposed directional protection detection algorithm.

**Table 7:** Changing the source impedance PPS behavior (scenario 16).

The conditions studied					Results	
Impedance Z_1SA_ (Ω)	Impedance Z_1SB_ (Ω)	Fault types	Fault position	Resistance (Ω)	Z matrix value	Direction result
0.72 + J8.2	1.4 + J1.6	AG	F	20	-1.9	F
0.72 + J8.2	1.1 + J1.3	AG	R	30	3.5	R
0.09 + J2.5	1.3 + J1.5	BG	F	15	-1.6	F
0.09 + J2.5	1.4 + J.6	BG	R	5	2.5	R
0.59 + J6.7	1.1 + J1.3	CG	F	15	-4.2	F
0.59 + J6.7	1.3 + J1.5	CG	R	20	3.6	R
0.72 + J8.2	1.4 + J1.6	ABG	F	35	-3.8	F
0.72 + J8.2	1.1 + J1.3	ABG	R	15	3.9	R
0.09 + J2.5	1.3 + J1.5	ACG	F	5	-2.6	F
0.09 + J2.5	1.4 + J1.6	ACG	R	10	5.9	R
0.59 + J6.7	1.1 + J1.3	BCG	F	15	-1.8	F
0.59 + J6.7	1.3 + J1.5	BCG	R	30	4.4	R
0.72 + J8.2	1.4 + J1.6	AB	F	20	-3.3	F
0.72 + J8.2	1.1 + J1.3	AB	R	5	3.1	R
0.09 + J2.5	1.3 + J1.5	BC	F	24	-3.2	F
0.09 + J2.5	1.4 + J1.6	BC	R	38	2.8	R
0.59 + J6.7	1.1 + J1.3	AC	F	5	-2.5	F
0.59 + J6.7	1.3 + J1.5	AC	R	15	5.6	R
0.09 + J2.5	1.4 + J1.6	ABCG	F	26	-3.7	F
0.72 + J8.2	1.1 + J1.3	ABCG	R	1	3.3	R
0.59 + J6.7	1.3 + J1.5	ABC	F	18	-2.9	F
0.09 + J2.5	1.4 + J1.6	ABC	R	10	4.7	R

#### Influence of source strength on the PPS: scenario 17

If the short-circuit is not adequate to initiate protective relays. The behavior of the proposed directional protection detection algorithm is analyzed in such conditions (weak in-feed). In this scenario, Table 8 shows the results that confirms the robustness and effectiveness of the proposed directional protection detection algorithm.

**Table 8:** Large source impedance PPS behavior (scenario 17).

The conditions studied				Results	
Short circuit level	Fault types	Fault position	Resistance (Ω)	Z matrix value	Direction result
1500 MVA	ABG	F	10	-1.4	F
	AG	F	20	-2.5	F
	ACG	F	18	-3.1	F
	BG	F	40	-4.5	F
	AC	F	15	-1.7	F
	BC	F	35	-2.6	F
1000 MVA	ABG	F	25	-2.3	F
	BCG	F	30	-4.5	F
	ACG	F	5	-3.4	F
	ABC	F	35	-3.3	F
	AB	F	25	-2.8	F
2000 MVA	ABG	R	20	2.5	R
	BCG	R	25	3.8	R
	AC	R	5	3.6	R
	AC	R	1	2.6	R
	AB	R	40	4.5	R
	AB	R	1	2.7	R
800 MVA	ABG	R	5	4.1	R
	BCG	R	25	3.4	R
	ACG	R	10	4.6	R
	ABC	R	35	4.7	R
	AB	R	5	3.8	R

#### Influence of different transmission line length on PPS: scenario 18

The proposed directional protection detection algorithm performance has been investigated for different line length in case of double circuit system. Firstly, the line length was 140 km. Moreover, the proposed directional protection detection algorithm examined under dissimilar line length as shown in Table 9. Thus, the proposed directional protection detection algorithm has not affected by this scenario.

**Table 9:** PPS behavior under dissimilar transmission line length (scenario 18).

The considered conditions				Results	
Line length (Km)	Fault types	Fault position	Resistance (Ω)	Z matrix value	Direction result
70	AG	F	1	-2.4	F
140	AG	R	40	2.5	R
140	ABG	F	25	-3.8	F
200	ABG	R	20	3.2	R
200	AB	F	100	-2.8	F
80	ABCG	R	35	2.9	R
100	ABCG	F	25	-4.1	F
120	CG	F	0.5	-3.7	F
180	CG	R	1	3.8	R
160	BCG	F	50	-4.6	F
70	BCG	R	100	4.8	R
80	AC	F	0.5	-3.4	F
200	AC	R	0.5	3.5	R
80	ABCG	F	13	-4.2	F

#### Influence of CT saturation on PPS: scenario 19

**Fig. 17:**
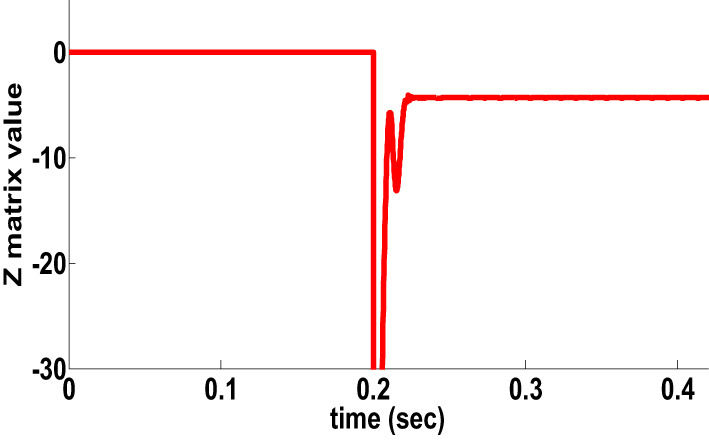
CT saturation result for single circuit line (ABCG fault).

The performance of proposed scheme for the case of current transformer (CT) errors is evaluated here. The heavy fault current in transmission line with decaying DC offset causes CTs saturation which are connected between both the ends of the protective zone. The secondary currents of saturated CTs are reduced to very low value as compare to actual value. The situation makes maloperation in most of the relaying algorithms. To evaluate the performance of the proposed algorithm for CT saturation, a three-phase fault in a single circuit is simulated beyond bus-L (forward). At bus R the CT is designed with magnetization characteristic as in^55^. As well as testing the impact of CT saturation, the secondary resistive burden of CTs is increased from 0.5 to 2.5 Ω. The measured fault current signal at CT having nonlinear characteristics. The computed line resistance is plotted in Fig. 17 using (11) and the negative value depicts during forward fault the decision of the proposed algorithm on direction computation is correct and not affected by CT saturation.

## Generality of PPS

**Fig. 18:**
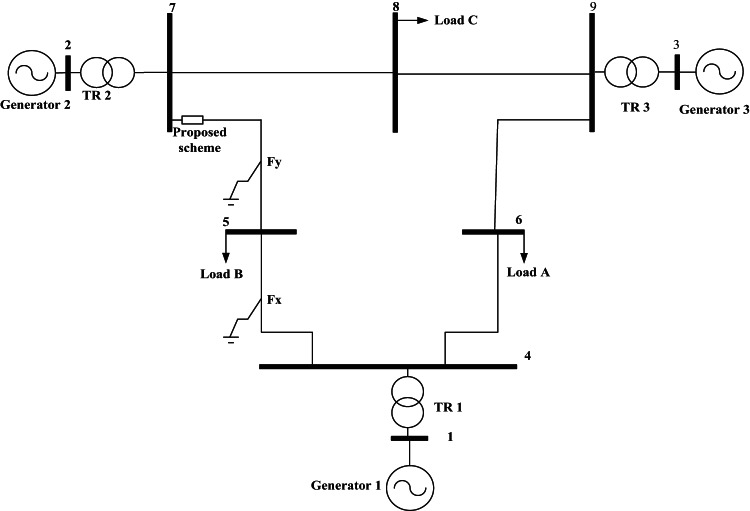
WSCC 9 bus system.

**Fig. 19:**
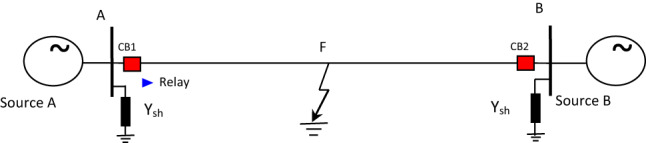
Single line diagram for transmission system model equipped with shunt reactors.

The proposed directional protection detection algorithm is comprehensive for any transmission systems. Consequently, proposed directional protection detection algorithm is utilized in a WSCC 3-machine power system, a 9-bus configuration shown in Fig. 18. Also, to assess the proposed protection scheme in a more realistic power system, this generalization was done. WSCC 9 bus system parameters are given in^38^. The proposed directional protection detection algorithm is mounted between buses 7 and 8 as shown in Fig. 18. Table 10 shows the results that confirm the robustness and effectiveness of the proposed directional protection detection algorithm. Also the proposed protection scheme can be extended for any transmission system As a part of generalization, a simulation study is conducted using a 500 kV, 50 Hz transmission system model equipped with shunt reactors, as depicted in Fig. 19. The simulation work is conducted using ATP/EMTP software. The line parameters are provided in^56^.

The proposed directional protection detection algorithm behavior in case of different fault parameters (types, fault location, fault time, fault resistance, load angle) are presented for transmission system model equipped with shunt reactors. The results of different fault parameters and fault conditions are confirmed in Table 11. It is spotted that the proposed directional protection detection algorithm decision has high precision as per Table 11 under different fault conditions.

## Dependability and security of PPS

Moreover, the reliability and sensitivity of the proposed scheme with healthy disturbance was also tested by varying source impedances, varying operating voltage, varying operating frequency, noise effect, different sampling frequency and different transmission line lengths. In addition, the response of the proposed scheme based on dependability and security were discussed and the results obtained showed that the proposed scheme has a good performance.

The proposed scheme is appraised using two significant characteristics: dependability and security. Basically, it is necessary that the proposed scheme provides the protective relay including the capability to achieve both dependability and security without compromising either. In this case some false tripping may happen, which can lead in the worst case to catastrophic impact on power system. In fact, the dependability indicates to a certainty grade that proposed scheme will run correctly, and this term is mainly dependent on the relay sensitivity. To demonstrate the performance of the proposed scheme, the reliability, dependability and security of the proposed scheme are measured as

15 $$\:\text{\%}\text{R}\text{e}\text{l}\text{i}\text{a}\text{b}\text{i}\text{l}\text{i}\text{t}\text{y}\:=\left(\frac{No\:of\:correct\:trip}{Total\:no\:of\:desired\:trip+No\:of\:incorrect\:trip}\right)*100$$

16 $$\:\text{\%}\text{D}\text{e}\text{p}\text{e}\text{n}\text{d}\text{a}\text{b}\text{i}\text{l}\text{i}\text{t}\text{y}=\left(\frac{No\:of\:correct\:trip}{Total\:no\:of\:desired\:trip}\right)*100$$

17 $$\:\text{\%}\text{S}\text{e}\text{c}\text{u}\text{r}\text{i}\text{t}\text{y}\:=\left(\frac{No\:of\:correct\:trip}{Total\:no\:of\:trip}\right)*100$$

Considering all studied cases based on fault parameter variations,

It was found that the relay operated 200 times, out of which 196 were correct trips. If the relay failed to issue trip decision on **2** situations; therefore, the measured dependability of the proposed scheme is 98.9%. Meanwhile, the term of security is key feature in a protective relay in which it measures the certainty grade that the relay will not run incorrectly. Furthermore, the security term is mainly dependent on the relay selectivity. To clarify the performance of proposed scheme based on the security term, the security formula is expressed by (17) therefore, the measured security of the proposed scheme is 98%. Finally, the measured reliability of the proposed scheme is 97%. Numerous healthy and unhealthy disturbances have been examined to prove the efficacy of the proposed scheme for different fault parameters. The results showed the ability of the proposed scheme to identify the fault direction.

## Behavior evaluation of distinctive schemes

**Fig. 20:**
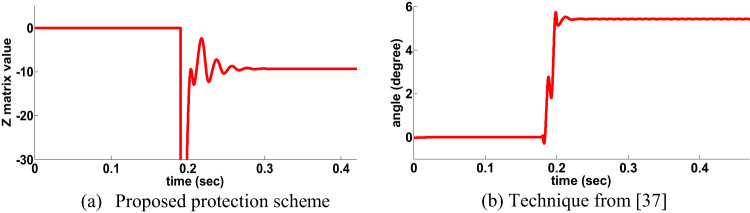
Behavior for a CG fault (210 Ω).

Performance evaluation study between the proposed directional protection detection algorithm and other schemes in the literature is presented in Table 12. The proposed directional protection detection algorithm does not need any compensation schemes in case of PFC. Ref^37^. utilized the phase angle differentiation between currents but it affected by HFR. This scheme examined similar situations to the same scenario in this paper, a CG fault type occurred that has a fault resistance of 210 Ω on the forward side for single circuit line. The Z matrix value of the PPS was estimated, and it was given the negative value for a forward side fault, as in Fig. 20.a. The angle difference was given the positive with the presence of fault resistance, as shown in Fig. 20.b. The PPS delivers a correct decision during HFR while the scheme in Ref^37^. is fails during same HFR.

**Table 10:** Effect of fault types, fault location, fault inception time and fault resistance for WSCC 9 bus system.

The considered conditions				Results	
Fault types	Fault location	Resistance (Ω)	Inception Instant (sec)	Z matrix value	Direction result
AG	F	1	0.156	-2.4	F
AG	R	20	0.156	2.8	R
CG	F	100	0.183	-2.9	F
BG	R	60	0.166	3.4	R
ACG	F	25	0.174	-3.7	F
CG	R	1	0.131	3.5	R
AB	F	1	0.152	-1.9	F
ABG	R	100	0.162	2.8	R
AC	F	25	0.184	-2.8	F
ACG	R	15	0.198	2.7	R
ABC	F	0.5	0.169	-3.5	F
BCG	R	35	0.182	3.8	R
BG	F	50	0.192	-3.5	F
AB	R	0.1	0.171	4.1	R
ABG	F	0.5	0.191	-4.2	F
BC	R	1	0.175	2.7	R
BCG	F	30	0.168	-2.4	F
AC	R	50	0.177	3.8	R
BC	F	100	0.165	-3.4	F
ABCG	R	100	0.183	2.8	R
ABCG	F	1	0.158	-3.7	F
ABC	R	0.5	0.171	4.2	R

Commercial protective relay ABB 670 series in^57^. is compared with PPS. By comparing the main key for both ABB 670 protective relay and PPS, ABB 670 is depended on DFT and PPS is depended on Z matrix value. DFT is assumes the fault signal is a stationary signal, lead to eliminate the time-frequency information which consider the major drawback in using its algorithm. Practically, the signals comprise numerous non-stationary signals. In^58^, the Fourier analysis is not suitable for detecting the transitory characteristics.

**Table 11:** Influence of different fault parameters (types, fault location, fault time, fault resistance, load angle) for transmission system model equipped with shunt reactors.

The considered conditions					Results	
Fault types	Fault position	Resistance (Ω)	Inception Instant (sec)	Load angle of sources (degree)	Z matrix value	Direction result
AG	Forward	10	0.18	5	-3.2	Forward
AG	Reverse	8	0.19	10	4.5	Reverse
CG	Forward	5	0.15	8	-6.2	Forward
CG	Reverse	50	0.17	9	4.9	Reverse
ACG	Forward	11	0.14	11	-3.9	Forward
ACG	Reverse	15	0.16	12	5.2	Reverse
AB	Forward	10	0.18	5	-6.8	Forward
AB	Reverse	25	0.16	4	4.2	Reverse
BC	Forward	30	0.14	6	-3.8	Forward
BC	Reverse	5	0.17	3	5.7	Reverse
ABCG	Forward	1	0.15	10	-6.4	Forward
ABCG	Reverse	0	0.19	12	4.9	Reverse
ABC	Forward	9	0.16	5	-7.0	Forward
ABC	Reverse	15	0.14	9	3.8	Reverse
BC	Forward	25	0.15	8	-5.7	Forward
BC	Reverse	2	0.18	6	4.6	Reverse
AC	Forward	10	0.17	7	-5.8	Forward
AC	Reverse	40	0.14	4	6.4	Reverse
BCG	Forward	5	0.16	6	-5.7	Forward
BCG	Reverse	30	0.18	9	5.2	Reverse
ACG	Forward	50	0.15	8	-4.6	Forward
ACG	Reverse	1	0.17	10	5.0	Reverse

**Table 12:** Performance assessment of distinctive schemes discussed in the literature.

Item ======= Scheme	Technique used	Features calculation	Double/ Single Circuit	HFR	PFC Detect
Ref.^11^	DFT	Pre-fault and fault current	Distribution system	Success	No need
Ref.^12^	LSM	PSC voltage (Superimposed) & PSC current (Superimposed)	Single circuit only	Success	Need
Ref.^23^	DFT	Fundamental components of voltage and current	Single circuit only	Success	Need
Ref.^25^	LSM and VT	Four Indices (Ind_1_-Ind_4_)	Double circuit only	Success	Need
Ref.^27^	Component analysis and LSM	(Ind_1_-Ind_3_) Three Indices	Double circuit only	Success	Need
Ref.^36^	Component Energy	Current and voltage fundamental components	Distribution system	Success	No need
Ref.^37^	DFT	NSSC current	Single & double circuit2	Failure	Need
Ref.^38^	DFT	NSSC current and voltage	Single circuit only	Success	Need
PPS	Samples only	Positive-sequence impedance	Single & double circuit2	Success	No need

The innovation of the proposed directional protection detection algorithm and the greatest significant characteristics are informed in the following points:

- Proposed directional protection detection algorithm relies on the single end measurement (no need for communication schemes) that decreases the cost.
- Travelling wave-based DR is negligible compared to sampling frequency for PPS. • The robustness of the improved directional protection scheme depends on the Z matrix value to avoid false action during noise effects, different transmission lines, SC and SPT.
- Proposed directional protection detection algorithm can be utilized small sampling frequency that is smaller than the sampling frequency required to apply the travelling wave.
- The Proposed directional protection detection algorithm doesn’t required knowledge of the fault ignition time.
- The Proposed directional protection detection algorithm is characterized by very fast response and precise measurements.
- Additional artificial intelligence techniques are not needed for support in the PPS, resulting in a simplified scheme compared with the competitive schemes in the literature.
- Improved directional protection scheme depends on positive sequence impedance component and avoids false action during PFC.

## Conclusion

Protection of TL systems present several challenges due to the unique characteristics. Fault direction scheme is greatest significant schemes used to protective them. The traditional schemes for determine the fault direction have issues in particular circumstances such as HFR, SPT and PFC. In this article, a novel directional protective scheme is proposed for single and double TL. A new directional protection detection algorithm is proposed based on the calculated nominal value of the voltage and current signals that utilized to get the Z matrix value. Through extensive testing on a simulated TL, the proposed protection technique exhibited reliable performance across various fault scenarios, including fault types, locations, high impedances, cross-country fault, inter-circuit fault, far-end fault, CT saturation, SPT, PFC, noise effect, variation in operating voltage, variation in source strength and different sampling frequency. The results indicate that the proposed approach competently classifies the direction of fault. Assessment with other published schemes declare the precision and suitability of the proposed directional protection detection algorithm.

Finally, we should point out that other factors that were beyond the framework of the study, and will be included in future studies, are considering renewable energy sources, the power quality issues, cables, combination between combination and transmission lines and studying the behavior of the proposed scheme in case of power swing condition.

## Data Availability

The authors confirm that the data supporting the findings of this study are available within the article Data Availability Statement: All data generated or analysed during this study are included in this published article.

## List of symbols

${I}_{1}{\prime}$: Superimposed positive sequence current component
${V}_{1}{\prime}$: Superimposed positive sequence voltage component
${\text{E}}_{\text{A}}$: The A’s equivalent positive-sequence voltage
${\text{E}}_{\text{B}}$: The B’s equivalent positive-sequence voltage
${\text{I}}_{1\text{ f}}$: Positive sequence current component at the relay location with fault
${\text{I}}_{1\text{ pre}}$: Positive sequence current component at the relay location without fault
${\text{V}}_{1}^{\text{f}}$: Positive sequence voltage component at the relay location with fault
${\text{V}}_{1}^{\text{pre}}$: Positive sequence voltage component at the relay location without fault
*I*: Phase A current
*I*_*b*_: Phase B current
*I*_*c*_: Phase C current
*Ind1*: Phase angel of positive sequence components
Ind2: Phase angel of superimposed positive sequence currents components
Ind3: Phase angel of superimposed positive sequence current and voltage components
Ind4: Phase angel of superimposed negative sequence current and voltage components
I_SPT1_: Positive sequence current component during single pole tripping
*S*_*f*_: Sampling frequency
*V*_*a*_: Phase A voltage
*V*_*b*_: Phase B voltage
*V*_*c*_: Phase C voltage
V_SPT1_: Positive sequence voltage component during single pole tripping
Z_PM1_: Positive sequence impedance of line AB
ZMN1: Positive sequence impedance of line BC

## Abbreviations

ANFIS: Adaptive neuro-fuzzy inference system
ANN: Artificial neural network
CM: Commutative method
CT: Current transformer
DFT: Discrete fourier transform
DWT: Discrete wavelet transform
F: Forward direction
FL: Fuzzy logic
FSM: Finite state machine
GPT: General park transformation
HFR: High fault resistance
LST: Least square technique
n: Number of sample
N.R: Not reported
NSC: Negative sequence component
NSSC: Negative-sequence-superimposed component
PCA: Principal component analysis
PFC: Power flow change
PFD: Power flow direction
PPS: Proposed protection scheme
PSC: Positive sequence component
R: Reversed direction
SPSC: Superimposed positive sequence component
SPT: Single pole tripping
SVM: Support vector machine
TL: Transmission line
TW: Travelling wave
VT: Voting technique

## Symbols

−: Subtract
&: Ampersand
*: Multiplication sign
[]: Brackets
+: Addition
=: Equality
> , <: Is less than, is large than
J: Complex number
$${e}^{x}$$: Exponential function
Not gate
And gate
Or gate
